# Competing endogenous RNA networks: tying the essential knots for cancer biology and therapeutics

**DOI:** 10.1186/s13045-015-0129-1

**Published:** 2015-03-28

**Authors:** Avencia Sanchez-Mejias, Yvonne Tay

**Affiliations:** Cancer Science Institute of Singapore, National University of Singapore, Singapore, 117599 Singapore; Department of Biochemistry, Yong Loo Lin School of Medicine, National University of Singapore, Singapore, 117597 Singapore

**Keywords:** microRNA, ceRNA, Competing endogenous RNA, Competing endogenous RNA networks, miRNA/ceRNA ratio, Cancer therapy, miRNA-based therapy

## Abstract

A recently discovered dimension of post-transcriptional gene regulation involves co-regulatory crosstalk between RNA transcripts, which compete for common pools of microRNA (miRNA) molecules. These competing endogenous RNAs (ceRNAs), or natural miRNA sponges, have an active role in regulating miRNA availability within the cell and form intertwined regulatory networks. Recent reports have implicated diverse RNA species including protein-coding messenger RNAs and non-coding RNAs as ceRNAs in human development and diseases including human cancer. In this review, we discuss the most recent discoveries that implicate natural miRNA decoys in human cancer biology, as well as exciting advances in the study of ceRNA networks and dynamics. The structure and topology of intricate genome-scale ceRNA networks can be predicted computationally, and their dynamic response to fluctuations in ceRNA and miRNA levels can be studied via mathematical modeling. Additionally, the development of new methods to quantitatively determine absolute expression levels of miRNA and ceRNA molecules have expanded the capacity to accurately study the efficiency of ceRNA crosstalk in diverse biological models. These major milestones are of critical importance to identify key components of ceRNA regulatory networks that could aid the development of new approaches to cancer diagnostics and oligonucleotide-based therapeutics.

## Introduction

MicroRNAs (miRNAs) comprise a class of small non-coding RNAs implicated in post-transcriptional RNA regulation. These RNA molecules are approximately 22 nt in length and bind to miRNA response elements (MREs) on target transcripts through sequence complementarity, usually inducing repression or destabilization of the target transcript [1,2]. A single miRNA can regulate up to thousands of target transcripts [3], and miRNAs can act in a combinatorial manner by binding to separate MREs on a single RNA transcript. MiRNA-mediated regulation is one of the most widespread post-transcriptional regulatory mechanism in eukaryotes, and is estimated to affect the majority of human transcripts [4,5]. In fact, accumulating evidence highlights the role of miRNA-mediated regulation in cell growth, differentiation, proliferation, and apoptosis [6]. Furthermore, miRNAs have been implicated in many diseases including cancer [7,8]. Alterations in the miRNA balance in the cell can lead to dysregulation of tumor suppressor genes and/or oncogenes controlled by aberrantly expressed miRNAs, leading to cancer [9,10].

Although the miRNA field is relatively young, a miRNA-based therapeutic has already entered phase 2 clinical trials [11,12]. In cancer, therapeutic strategies targeting miRNAs can be divided into two opposing approaches: (1) MiRNA mimics, which restore the function of tumor suppressive miRNAs; and (2) MiRNA inhibitors, which antagonize the function of oncogenic miRNAs. This can be achieved either by using chemically-modified RNA oligos which mimic or inhibit miRNA function or by artificially generated ‘miRNA sponges’ [13-15]. These exogenously expressed transcripts contain tandem repeats of MRE sites that allow them to specifically bind a distinct miRNA or combination of miRNAs. Once the miRNA(s) of interest has been decoyed, it will be unavailable to bind to its targets, leading to the effective de-repression of these transcripts [13,16].

In a similar fashion to how artificial miRNA sponges regulate the expression of endogenous miRNAs, it has been recently demonstrated that endogenous transcripts which contain MREs can interact and co-regulate each other through competition for common miRNA pools in the cell, acting as endogenous miRNA sponges or competing endogenous RNAs (ceRNAs) [17,18]. This intricate interplay between diverse RNA species, whereby one transcript can reciprocally modulate the expression of another transcript by sequestering shared miRNAs, has been referred to as ceRNA crosstalk [19]. CeRNA activity has been attributed to both protein-coding and non-coding RNA transcripts. Examples of this competing endogenous crosstalk have been described in plants, such as *Arabidopsis thaliana* [20], the primate virus *Herpesvirus saimiri* [21], zebrafish [22], and mice as well as humans [23,24].

The implication of ceRNAs in humans has been extended from normal cell differentiation to tissue development and pathology, since relevant ceRNA functionality has been associated with the self-renewal capacity of embryonic stem cells [25], muscle differentiation [23], idiopathic pulmonary fibrosis [26], and liver cancer [27] among other biological processes. Although ceRNA research is in its infancy, accumulating evidence suggests that this additional dimension of post-transcriptional gene regulation, in which RNA transcripts titrate miRNA availability, represents a biologically relevant, well-conserved and widespread mechanism of regulation in eukaryotes.

## Competing endogenous RNAs in cancer

**Figure 1:**
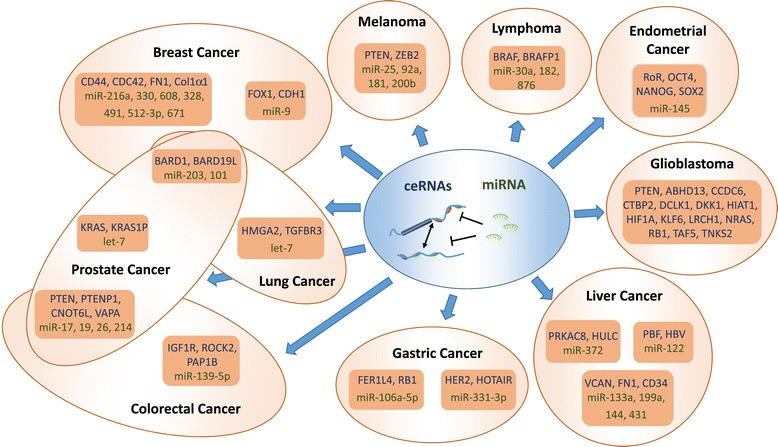
**Involvement of ceRNA-mediated regulation in human cancers**. Schematic representation of a simplified ceRNA network with two transcripts and one miRNA (blue circle), and the different cancers for which ceRNA activity has been experimentally verified (orange circles). Different ceRNA subnetworks are represented by squares, where ceRNAs (in blue) interact among each other by binding and competing for common miRNA molecule pools (in green). Validated ceRNA interactions have been reported for breast cancer [39,72-74], melanoma [35], endometrial cancer [38], glioblastoma [36], liver cancer [27,74,75], gastric cancer [40,60], colorectal cancer [17,34,76], prostate cancer [17,34,39], lung cancer [39,55], and lymphoma [41].

Several examples of MRE-containing non-coding RNA transcripts acting as ceRNAs have been described. This suggests that many long non-coding RNAs may potentially function as ceRNAs, and that the analysis of ceRNA interactions may facilitate the functional characterization of such non-coding transcripts. Interestingly, there is a widespread prevalence of somatic mutations, gross genetic alterations, and chromosome rearrangements affecting the non-coding genome in cancer [28,29]. Additionally, cancer cells often express alternative isoforms of mRNAs with shorter 3′UTR regions [30]. These events may lead to the mutation or loss of MREs, which will not only affect miRNA regulation *in cis* but also the associated ceRNA interactions. The dysregulation of ceRNA interactions may, in turn, have profound implications for cancer initiation, maintenance, or progression. Thus, it is perhaps unsurprising that significant research efforts thus far on ceRNA-mediated post-transcriptional regulation have focused on cancer initiation, maintenance, or progression (Figure 1). The role of ceRNAs in cancer biology has been reviewed in detail elsewhere [19,31]. As such, in this review, we will focus mainly on the most recent developments in the field.

The most extensively characterized ceRNA network (ceRNET) is perhaps that of the tumor suppressor gene *PTEN.* The processed pseudogene *PTENP1,* which is highly homologous to *PTEN* and shares many of its 3′UTR MREs, was the first non-coding transcript shown to post-transcriptionally regulate *PTEN* expression in a miRNA-dependent manner [17]. In a recent study, *PTENP1* was reported to be downregulated in clear-cell renal cell carcinoma and shown to regulate PTEN expression. *PTENP1* tumor suppressive properties were due in part to sequestration of miR-21, providing additional support for the functional relevance of *PTENP1* as a tumor suppressive ceRNA for PTEN [32]. Additional studies have identified various mRNA transcripts that function as PTEN ceRNAs and regulate PTEN expression in a 3′UTR and miRNA-dependent manner in brain, breast, colon, prostate, and skin cancers [33-36].

Recent reports have provided increasing support for the ceRNA activity of long non-coding RNAs (lncRNAs), suggesting that the characterization of ceRNA networks may enable researchers to systematically study the function of these largely uncharacterized RNA transcripts. In addition, lncRNAs often display specific expression patterns in different cell types, tissue types, developmental stages, and disease [37], making them ideal candidates for participating in finely tuned post-transcriptional regulation.

One such example is linc-RoR, which had already been shown to control self-renewal and maintain pluripotency of human embryonic stem cells by acting as a miR-145 ‘sponge’ and thus controlling OCT4, NANOG, and SOX2 expression [25]. Similarly, linc-RoR was found to regulate expression of the same transcription factors in endometrial cancer stem cells, and inhibit their differentiation, in a miR-145 dependent manner [38].

Another example of lncRNA-mediated ceRNA regulation involves the tumor suppressor gene *BARD1*. The newly discovered lncRNA *BARD1 9′L* is transcribed from an alternative intronic promoter of the *BARD1* gene, and was found to share both miR-203 and miR-101 MREs with *BARD1* mRNA in their homologous 3′UTRs [39]. Moreover, full-length *BARD1* and cancer-associated *BARD1* mRNAs were downregulated by miR-203 and miR-101, and lncRNA *BARD1 9′L* counteracted the effect of these miRNAs. These data support a role for *BARD1 9′L* as a tumor suppressor transcript through its ceRNA activity.

Conversely, the ceRNA activity of lncRNAs has also been shown to have an oncogenic effect: The lncRNA HOTAIR was shown to display ceRNA activity in gastric cancer cells, in which it was found to specifically bind the tumor suppressor miR-331-3p, modulating HER2 de-repression [40]. In addition, the authors observed an association of HOTAIR expression with cancer progression and malignancy in clinical samples from patients with advanced gastric cancer.

A very recent report explored the role of the *BRAF* pseudogene (*BRAFP1*) in regulating the expression of *BRAF* in a miRNA-dependent manner [41]. This study demonstrated that *BRAFP1* functions as a ceRNA for *BRAF* in part by sponging miR-30a, miR-182, and miR-876, and that the oncogenic effect of *BRAFP1* overexpression *in vitro* was at least partly mediated by the upregulation of BRAF expression and subsequent activation of MAPK. Moreover, the authors presented a mouse model overexpressing full-length murine *B-Raf* pseudogene that developed an aggressive malignancy resembling human diffuse large B cell lymphoma. This study is the first one to demonstrate the oncogenicity of a pseudogene in an engineered mouse model, and provides further *in vivo* support for the role of ceRNA-mediated miRNA sequestration on cancer development.

## Molecular considerations for ceRNA interactions

Although there are increasing reports describing *bona fide* ceRNA interactions in diverse biological settings, several open questions remain about the physiological relevance of these interactions [42]. Factors such as miRNA- and ceRNA-expression levels and subcellular localization, number of shared MREs, and miRNA/ceRNA binding affinity have all been suggested to modulate the effectiveness of ceRNA crosstalk [18,43,44]. Indeed, it has been shown that molecular interactions that are dependent on titration mechanisms, such as ceRNA crosstalk, usually display a threshold-like behavior determined by the relative abundance of interacting molecules [45].

Several recent reports have described various computational models that have shed light on the specific molecular requirements for effective ceRNA interactions both at steady-state conditions, and in dynamic conditions when the system transiently responds to perturbations around the threshold level [46-50]. Using an *in silico* mathematical mass-action model to determine the optimal conditions for ceRNA interactions, Ala et al. identified several molecular determinants for effective ceRNA crosstalk [46]. These determinants include factors such as miRNA and ceRNA expression levels, the number of MREs shared between ceRNAs, and the total number of MREs for the miRNAs of interest. In an alternative approach, Figliuzzi et al. utilized a minimal rate-equation-based model of post-transcriptional regulation at steady-state levels to characterize the nature of ceRNA interactions [48]. Interestingly, they suggest that ceRNA interactions can be either symmetrical, whereby two ceRNAs co-regulate each other, or asymmetrical, whereby one ceRNA regulates expression of the other in a unidirectional manner. In addition to steady-state system assumptions, researchers have harnessed various mathematical models to investigate the dynamics of ceRNA crosstalk. Studies have described the equilibrium phenomenon that the system displays in the threshold proximity that is found at near equimolar conditions [47], as well as the transient response of the system to perturbations [49]. In addition, a recent report presented a computational model to quantitatively describe a minimum ceRNA network, and experimentally validated the predictions in cultured human cells using synthetic gene circuits [50].

**Figure 2:**
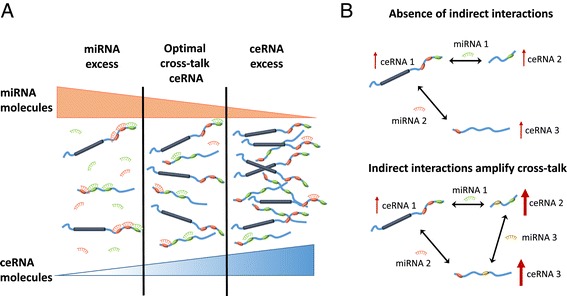
**Molecular dynamics of ceRNA interactions. (A)** Optimal molecular conditions for effective ceRNA crosstalk depend on miRNA/ceRNA ratios. Schematic representation of a simplified ceRNA network involving two transcripts (with blue backbones) harboring two different MRE sites (orange and green circles) that bind to miRNA1 (orange) and miRNA2 (green), respectively. Variations in ceRNA expression levels would generate a response in the expression of co-regulated ceRNAs only in a narrow miRNA concentration window, and only for a range of ceRNA concentration. When miRNA concentration is in excess, the titration capacity of competing MRE is diminished and no effect would be observed on ceRNA crosstalk. Conversely, in excess of ceRNA, most miRNA molecules will bind their MRE sites, and the system would be insensitive to changes in the relative miRNA/ceRNA ratios. **(B)** The presence of indirect interactions can amplify ceRNA crosstalk. Schematic representation of two ceRNA networks involving three ceRNAs and two (top panel) or three (bottom panel) miRNAs. When ceRNA 2 is able to co-regulate ceRNA 3 by sequestering miRNA 3, the effect of ceRNA 1 upregulation on ceRNA 2 and ceRNA 3 levels may be amplified by this secondary crosstalk. This is termed an indirect interaction as it does not involve a direct relationship between ceRNA 1 and either ceRNA 2 or ceRNA 3.

Despite the differences in the mathematical approaches, interaction parameters, and assumptions for these studies, several common conclusions can be drawn. For instance, optimal ceRNA-mediated regulation was observed to depend heavily on the miRNA/ceRNA ratio [46,47,50], consistent with quantitative experimental data demonstrating that the optimal conditions for miRNA functionality depended largely on miRNA/target relative abundance [50,51]. Optimal ceRNA crosstalk, whereby one ceRNA has the most striking effect on its interacting ceRNA partners, was predicted to occur when miRNA and ceRNA levels were near equimolarity [47,51] (Figure 2A). Another common finding was that the number of shared miRNAs among RNA transcripts played an important role in determining the effectiveness of ceRNA crosstalk [46-48].

Interestingly, ceRNA crosstalk appeared to represent a viable mechanism to achieve fast, positive changes in transcript levels [49]. Additionally, mathematical models predicted that ceRNA crosstalk could represent a powerful noise-processing mechanism in gene expression that can lead to noise reduction or amplification [47,48]. These bioinformatic analyses suggest that ceRNAs may potentially play a central role in post-transcriptional regulation.

These mathematical models also led to some unexpected findings. For example, ceRNAs may interact with other ceRNAs directly and/or indirectly [46]. In the hypothetical network depicted in Figure 2B, ceRNA 1 interacts directly with ceRNA 2 via miRNA 1, and directly with ceRNA 3 via miRNA 2. In a situation where ceRNA 2 also interacts with ceRNA 3 via an additional miRNA 3 (Figure 2B, bottom panel), then ceRNA 1 would interact indirectly with ceRNA 2 via miRNA 2-ceRNA 3-miRNA 3. Ala et al. found that such indirect interactions were surprisingly effective at enhancing ceRNA crosstalk, and that ceRNAs were able to respond to changes in other ceRNAs even in the absence of shared miRNAs [46,47]. The relevance of indirect interactions to overall ceRNET architecture was corroborated by an independent study analyzing the interactions between distant ceRNET nodes in HEK293 cells [52]. Additionally, in optimal molecular conditions, ceRNETs may be altered by changes in the transcription rate of a single key ceRNA [47], and this effect is predicted to be greater for highly expressed ceRNAs [46]. These findings have enormous implications not only for the molecular study of cancer gene dysregulation but also for the exploration of miRNA sponges as new therapeutic tools.

As an essential complement to the above-mentioned analyses, which were largely computational, a number of recent studies have sought to experimentally quantify miRNA and target cellular abundance as well as miRNA target affinity. In a landmark study, a quantitative assessment of genome-wide miRNA regulation in the context of ceRNA crosstalk was performed in embryonic and mesenchymal stem cells, using Ago2 individual-nucleotide resolution UV crosslinking and immunoprecipitation, together with absolute miRNA and mRNA quantification [53]. They found that miRNA/ceRNA ratios determined not only Ago accumulation to its different affinity sites but also the susceptibility and threshold of miRNA-dependent ceRNA regulation. Single-cell miRNA reporter assays confirmed that low miRNA/ceRNA pool ratios, such as those found endogenously for the miR-92/25 family, possess an increased susceptibility for ceRNA regulation. On the other hand, highly expressed miRNA families such as miR-294 and let-7 were found to be insensitive to changes in relative ceRNA abundance in this experimental model. These data establish an experimental framework for the biochemical analysis of ceRNA interactions and provide critical experimental support for the physiological relevance of ceRNA crosstalk.

As discussed above, several computational and experimental studies have suggested that high abundance miRNAs may not be susceptible to effective ceRNA regulation. However, exceptions to this hypothesis may exist. The *HMGA2* mRNA was shown to decoy the highly abundant let-7 miRNA family, thus regulating TGFBR3 expression and enhancing TGF-β signaling [54]. This ceRNA activity of the *HMGA2* transcript was proposed to promote lung cancer progression. Critically, the authors found that *HMGA2* and *TGFBR*3 transcripts were expressed at similar levels, and that total let-7 family expression was within an order of magnitude of these transcripts in the lung cancer cell line tested. These data suggest that in certain settings, even highly abundant miRNAs such as let-7 may be effectively regulated by ceRNA interactions. Furthermore, the precise topology of ceRNETs may result in *bona fide* ceRNA regulation which may seem improbable based purely on specific miRNA/mRNA ratios. For example, mathematical modeling has predicted that effective interactions between two ceRNAs can be mediated by a large number of shared miRNAs, which may each only have a weak effect on ceRNA levels [48]. This suggests that future studies focused on high-throughput techniques to identify and characterize interconnected ceRNA networks will lead to important insights into the functional relevance of ceRNA interactions in complex physiological systems.

Indeed, the effectiveness of ceRNA crosstalk has been shown to vary in different physiological settings. For example, two recent studies examined the effect of sequestering the liver-specific miR-122. Denzler et al. analyzed the stoichiometric relationship between miR-122 and its targets in hepatocytes in both physiological and pathological conditions [55]. Due to the high expression levels of miR-122, they found that effective ceRNA crosstalk in this system could only be mediated by non-physiological increases in ceRNA levels and that the system was insensitive to up to threefold changes in the miRNA level. However, even though ceRNA crosstalk was lacking in physiological conditions for highly abundant miR-122, the system showed effective ceRNA interaction near the threshold where miRNA and target levels were near equimolarity, in agreement with the general consensus in the field. In contrast, a second study by Luna et al. described a quantitative mathematical model of miR-122 sequestration by the hepatitis C virus (HCV) RNA, and utilized HITS-CLIP and single-cell measurements to demonstrate that functional sequestration of miR-122 by the HCV RNA reduced miR-122 binding to its endogenous target mRNAs [56].

These studies present accumulating *in silico* and *in vitro* evidence indicating that the overall stoichiometry of the interacting molecules and miRNA/ceRNA binding affinity are critical determinants of effective crosstalk between ceRNAs *in vivo*. These factors should be considered when investigating novel ceRNA transcripts and networks, especially those of relevance to specific human disease conditions, to facilitate the successful translation of research results to clinical settings.

## Prediction of ceRNA networks in human cancer

Most ceRNA interactions reported to date have been between a pair of interacting transcripts. However, the ability of a single miRNA to bind and regulate multiple transcripts and the regulation of a single transcript by multiple different miRNA molecules allow the effects of relative changes in miRNA/ceRNA ratios to percolate through intertwined ceRNETs and affect the expression of distant ceRNAs. It is thus critical to begin to unravel the complexities of this regulatory crosstalk in genome-scale ceRNETs.

The availability of genome-wide miRNA-, mRNA-, and other transcript expression data in large cancer patient datasets, together with the development of novel algorithms and bioinformatic tools, enable the *in silico* prediction of ceRNA crosstalk. These predictions can be expanded, with the appropriate statistical treatment, to potentially generate genome-scale ceRNA network data for different biological processes, including cancer. Using this approach, breast and thyroid cancer-specific mRNA ceRNETs were recently described by two independent groups [57,58]. Although these results remain to be confirmed experimentally, they have proven useful to predict the risk of metastasis in breast cancer patients [57], and to highlight specific biological processes that may be central to certain cancers and regulated by ceRNA crosstalk, such as immune response regulation in papillary thyroid carcinoma [58].

Recently, a lncRNA-miRNA-mRNA ceRNET was predicted computationally in breast cancer patients [59]. This report found that ceRNETs may be significantly altered between normal and pathological breast tissues, and identified the lncRNA PVT1 as a key ceRNA on breast cancer. A second ceRNET analysis involving lncRNAs was described using genome-wide expression data from gastric cancer patients [60]. The authors integrated microarray data, bioinformatics, and a miRNA target database to generate a lncRNA-miRNA-mRNA network. Interestingly, this network contained a high number of mRNA targets implicated in cancer. Although relevant experimental validation will be needed to determine the implications of these findings, the authors showed co-regulation of the same ceRNAs in independent datasets from six other cancer types.

Recently, an intriguing new facet of ceRNET dysregulation in cancers was reported. Exome sequencing data from a large set of prostate cancer patients was analyzed to examine the effect of alternative cleavage on 3′UTRs and polyadenylation dynamics on putative ceRNET interactions [61]. Cancer patients could be stratified into groups with differing risks according to their alternative polyadenylation profiles, and a ceRNA subnetwork enriched with prostate cancer genes was found to be specifically dysregulated only in high-risk cancers. As 3′UTRs harbor multiple MREs which mediate miRNA binding, it is perhaps unsurprising that 3′UTR shortening can affect the associated ceRNETs. However, the observation that an unusually large number of MREs are shared between these significantly altered 3′UTRs and the affected ceRNA interactions suggests that ceRNETs may indeed be selectively dysregulated in high-risk prostate cancer.

The potential of studying ceRNA crosstalk in the context of larger interconnected networks, rather than isolated ceRNA pair interactions, opens exciting possibilities for the study of ceRNA-mediated post-transcriptional regulation in cancer in a setting that is closer to physiological and pathological conditions. However, there are still several considerations limiting the practical applicability of ceRNET study. One main limitation is the dependence of many ceRNA prediction tools on miRNA target prediction algorithms. Despite improvements in the field, these algorithms are not fool-proof due to our incomplete understanding of the rules for miRNA target recognition. This is particularly relevant for the prediction of mammalian miRNA and subsequent ceRNA networks, as mammalian miRNAs often pair with imperfect complementarity to their target transcripts. Although ceRNA prediction analysis has been successfully performed using validated miRNA interactors in the context of PTEN [34], miRNA target prediction tools were still required to identify binding of these validated PTEN-targeting miRNAs to potential ceRNAs of interest.

Additionally, given the scale of high-throughput data and the number of interactions taken into account, careful statistical treatment of the data needs to be performed for global ceRNET study. Moreover, the discovery of novel ceRNA interactions rely, in many cases, on the significant co-regulation of transcripts in cancer patient expression data. However, it has been reported that significant expression correlation among ceRNAs can emerge in experimental readouts due to transcriptional fluctuations even in the absence of miRNA-mediated crosstalk [48].

The development of high-throughput experimental validation of ceRNA interactions would thus be central to the expansion of our knowledge about ceRNETs and applicability for clinical practice in the future. Recent advances in techniques enabling the high-throughput sequencing of immunoprecipitated RNAs after crosslinking (such as CLIP-Seq, HITS-CLIP, and PAR-CLIP) provide a biochemical method to identify relevant miRNA target interactions [56]. For instance, the integration of HITS-CLIP data with computational approaches was shown to improve miRNA target prediction by over 20-fold [62]. An integrated database termed starBase v2.0 has been developed to identify experimentally supported regulatory RNA-RNA and protein-RNA interaction networks from multiple CLIP-Seq datasets [63]. starBase identified about 10,000 ceRNA pairs from CLIP-supported MREs, including miRNA-mRNA and miRNA-lncRNA interactions. As miRNAs, lncRNAs and even mRNAs often display tissue-, temporal-, and disease-specific expression patterns, such high-throughput biochemical analyses will be invaluable to characterize ceRNA interactions in specific cellular contexts.

## Conclusions

In recent years, diverse RNA species including mRNAs, pseudogenes, small non-coding RNAs, lncRNAs, and circular RNAs have all been shown to possess ceRNA activity in defined biological settings. Given the vast amount of genome-wide transcript expression data, and advances in the computational management of this publicly available data, we anticipate that reports of the ceRNA function of various RNA transcripts will continue to increase. Since miRNA decoying capacity is independent of protein-coding function, it has been proposed that the ceRNA activity of coding transcripts may allow them to exert independent and even opposing roles to their encoded protein in initiating or maintaining tumorigenic properties [18]. This in turn has important implications in the study, and potentially the clinical management, of human cancers. The existence of new ceRNA transcript classes also represents an exciting area for future study. Small RNA species such as rRNA and tRNA have been shown to interact with Argonaute proteins [64], raising the question of whether they could possess ceRNA activity.

In addition, ceRNA-mediated post-transcriptional regulation has opened the exciting possibility of methodically studying and functionalizing the non-coding transcriptome. In this respect, the identification of previously uncharacterized lncRNAs as ceRNA counterparts for mRNAs is flourishing. Interestingly, protein-coding genes that produce lncRNAs are over-represented in cancer association studies, being twice as likely to be associated to cancer as human protein-coding genes overall [65]. Moreover, specific regulatory patterns for several lncRNA transcripts have been found for a number of biological processes [37]. The ceRNA activity of non-coding transcripts may be enhanced by their escape from any form of interference from the translational machinery. All of this make lncRNAs great candidates for participating in finely tuned post-transcriptional regulation.

Most of the work done so far has focused on the study and quantification of key interacting RNA species such as miRNAs, mRNAs, lncRNAs, and other ceRNA transcripts. Future work investigating the potential interplay of ceRNAs with RNA binding proteins (RBPs) will provide further insights into the complexities of ceRNA crosstalk. Interestingly, the dynamic recruitment of Ago-interacting RNA-biding proteins has been shown to alter miRNA/target affinity [66,67]. A recent report has detailed the development of a database to identify potential RNA:RNA and RNA:protein regulatory interactions in multiple CLIP-Seq datasets encompassing miRNA, mRNA, lncRNA, circRNA, pseudogene, and RBP associations [63]. Experimental characterization of these RNA:RBP interactions will be needed to fully understand their impact on the dynamics of ceRNA interactions.

One of the most exciting advances in the ceRNA field in recent times has been the potential ability to computationally determine the structure and topology of miRNA and ceRNA interactions in interconnected ceRNETs, and harness mathematical modeling to study the dynamic behavior of these ceRNETs in response to fluctuations in the expression of ceRNAs and miRNAs in the network. Furthermore, the quantitative determination of absolute expression levels of miRNA and ceRNA molecules expands the capacity to accurately study the efficiency of ceRNA crosstalk in diverse physiological and pathological conditions. We anticipate that the systematic analysis of ceRNET dynamics would allow for the discovery of new oligonucleotide gene therapies, targeting highly connected nodes, which, unlike non-essential players in the network, may cause severe cellular damage when disrupted [68].

A better understanding of the molecular conditions in which ceRNA interactions occur is of critical importance to facilitate the study of this dimension of post-transcriptional regulation. Fluctuations in both miRNA and ceRNA levels *in vivo* need to be carefully examined to determine whether this dysregulation will lead to significant perturbation of ceRNA crosstalk in specific biological settings. The integration of this information with genome-scale ceRNET dynamics will be important to determine which ceRNAs are highly connected target hubs, less likely to be susceptible to compensation by alternative signaling pathways.

Multiple mechanisms may contribute to the dysregulation of miRNA and associated ceRNA interactions in cancer. For example, single nucleotide polymorphisms (SNPs) located in specific miRNA binding sites have been shown to modulate the binding of miRNAs to these target sites. A panel of 30 proto-oncogene associated SNPs that may impair miRNA target interactions was recently identified, and a subset of these was further associated with therapeutic outcome in cancer patients [69]. In addition to genomic alterations, sequence alterations at the transcript level such as those introduced through RNA editing have also been shown to effectively regulate miRNA target specification [70]. The contribution of such events to the dysregulation of ceRNA networks in cancer and potential subsequent impact on tumor progression and metastasis should be investigated in future studies.

In conclusion, the study of miRNA and ceRNA networks will open up new avenues for basic cancer research, as well as facilitate the development of novel diagnostic and therapeutic tools. For example, the silencing of aberrantly expressed miRNAs in cancer has been achieved through antisense oligomers, and recent advances in targeted delivery to tumor cells have shown promise in mouse models *in vivo* [71]. The identification of key miRNAs and ceRNAs in human cancer may thus represent promising new therapeutic targets. ceRNA network components could be targeted alone, to reduce oncogenic capacity of the cells, or in combination with traditional therapies to impair acquired drug resistance through compensatory pathways.
